# CRP, Fibrinogen, White Blood Cells, and Blood Cell Indices as Prognostic Biomarkers of Future COPD Exacerbation Frequency: The TIE Cohort Study

**DOI:** 10.3390/jcm13133855

**Published:** 2024-06-30

**Authors:** Jens Ellingsen, Christer Janson, Kristina Bröms, Maria Hårdstedt, Marieann Högman, Karin Lisspers, Andreas Palm, Björn Ställberg, Andrei Malinovschi

**Affiliations:** 1 Department of Medical Sciences, Respiratory, Allergy & Sleep Research, Uppsala University, 751 85 Uppsala, Sweden; 2 Department of Public Health & Caring Sciences, Family Medicine & Preventive Medicine, Uppsala University, 751 85 Uppsala, Sweden; 3 Center for Clinical Research Dalarna–Uppsala University, 791 82 Falun, Sweden; 4 Department of Medical Sciences, Clinical Physiology, Uppsala University, 751 85 Uppsala, Sweden

**Keywords:** COPD, acute exacerbation, systemic inflammation, white blood cells, CRP, fibrinogen

## Abstract

**Background/Objective**: Systemic inflammation is common in chronic obstructive pulmonary disease (COPD), and evidence suggests that inflammatory biomarkers can predict acute exacerbations (AECOPDs). The aim of this study was to analyse whether C-reactive protein (CRP), fibrinogen, white blood cell count (WBC), or the blood cell indices PLR (platelet-to-lymphocyte ratio), SII (systemic immune inflammation index), SIRI (systemic inflammation response index), and AISI (aggregate index of systemic inflammation) can predict future AECOPDs. **Methods:** In the Tools Identifying Exacerbations (TIE) cohort study, participants with spirometry-confirmed COPD were recruited from primary and secondary care in three Swedish regions and assessed during a stable phase of COPD. AECOPD frequency during the three-year follow-up was reviewed in medical records. Associations were analysed via ordinal logistic regressions. **Results:** Of the 571 participants, 46% had ≥1 AECOPD during follow-up, and the mean ± SD AECOPD frequency was 0.63 ± 1.2/year. In unadjusted analyses, high levels of CRP (odds ratio 1.86, 95% CI 1.29–2.67), fibrinogen (2.09, 1.38–3.16), WBCs (2.18, 1.52–3.13), SII (1.52, 1.05–2.19), SIRI (1.76, 1.23–2.52), and AISI (1.99, 1.38–2.87) were associated with a higher AECOPD frequency. After adjustment for AECOPD history, age, sex, smoking, body mass index, COPD Assessment Test score, lung function, and inhaled corticosteroid use, associations remained for high levels of CRP (adjusted odds ratio of 1.64; 95% CI of 1.08–2.49), fibrinogen (1.55; 1.07–2.24), and WBC (1.65; 1.10–2.47). **Conclusions:** CRP, fibrinogen, and WBC, assessed during stable-phase COPD, enhanced AECOPD prediction, whereas PLR, SII, SIRI, and AISI did not.

## 1. Introduction

Chronic obstructive pulmonary disease (COPD) is one of the major causes of morbidity and mortality worldwide [1]. Globally, the prevalence of COPD is estimated to be 10%, and about 3 million deaths every year are attributed to COPD [1]. These numbers are expected to increase, as the number of people smoking is increasing in many countries, and as populations age in other countries. Tobacco smoking and old age are significant risk factors for COPD. Others include exposure to indoor or outdoor air pollution, genetic factors, and premature birth [1]. Clinically, COPD is characterised by dyspnoea and chronic cough that often is productive. Fatigue is common, as is weight loss and sarcopenia at more severe stages of the disease.

A key feature of COPD is acute exacerbations (AECOPDs), i.e., episodes of worsening respiratory symptoms, which contribute substantially to the burden of COPD, with increased mortality, deterioration of lung function, and reduced quality of life [2,3,4]. Therefore, preventing AECOPDs is a crucial objective in the management of patients with COPD. Some patients never experience AECOPDs [5], whereas others do often. The frequent exacerbator phenotype is characterised by two or more AECOPDs yearly [6]. The strongest risk factor for future AECOPDs is a history of AECOPD [6], which means that patients risk falling into a self-reinforcing vicious circle of recurring AECOPDs. Other risk factors include older age, impaired lung function, a high burden of symptoms, underweight, and comorbidities [6,7].

Chronic inflammation of the airways is a key feature in the pathogenesis of COPD [8], and increased airway inflammation, often due to viral or bacterial infections, is a major mechanism in AECOPDs [9]. Systemic inflammation may also be present in COPD and is associated with worse outcomes, such as more frequent AECOPDs [10,11]. This insight has opened for inflammatory biomarkers analysed in peripheral blood, and significant scientific effort has been made to identify clinically relevant biomarkers in COPD. To date, however, the only biomarker from peripheral blood recommended in clinical routine is blood eosinophils to predict the effect of inhaled corticosteroids (ICS) in AECOPD prevention [1]. 

Systemic biomarkers that are suggested to be prognostic of future AECOPD include C-reactive protein (CRP), fibrinogen, and white blood cell count (WBC) [10,11,12], although their contribution compared to clinical data is small [11]. Therefore, there has been an increasing interest in composite biomarkers, integrating several markers into one, e.g., blood cells combined into indices or ratios such as the neutrophil-to-lymphocyte ratio (NLR) [13]. Other such blood cell indices suggested for AECOPD prognostics are the platelet-to-lymphocyte ratio (PLR), the systemic immune-inflammation index (SII, neutrophils × platelets/lymphocytes), and the systemic inflammation response index (SIRI, neutrophils × monocytes/lymphocytes), but only a few studies regarding these biomarkers and future AECOPDs have been published [14]. A novel index is the aggregate index of systemic inflammation (AISI, neutrophils × platelets × monocytes/lymphocytes), which has not been investigated in relation to AECOPDs before [15]. 

We previously investigated the prognostic role of eosinophils and the NLR, measured in a stable phase of the disease [16]. Now, we hypothesise that other biomarkers of systemic inflammation (CRP, fibrinogen, WBC, PLR, SII, SIRI, and AISI) measured during stable-phase COPD can predict future AECOPDs. 

## 2. Materials and Methods

### 2.1. Study Design, Setting and Participants

Tools Identifying Exacerbations in COPD (TIE) is a cohort study conducted at three study centres in the Swedish regions of Dalarna, Gävleborg, and Uppsala [17]. Participants with a physician’s diagnosis of COPD (International Classification of Diseases, 10th revision diagnosis code J44) were recruited from primary and secondary care between September 2014 and September 2016. All participants provided written informed consent. The Regional Ethics Review Board in Uppsala, Sweden, approved the study (Dnr 2013/358; final approval: 23 September 2015).

Inclusion criteria were a) a post-bronchodilator ratio of forced expiratory volume in one second (FEV_1_) to the highest of the slow vital capacity (SVC) and forced vital capacity (FVC) < 0.7 at the baseline visit, (b) age ≥ 40 years, (c) an ability to fill out questionnaires, and (d) an ability to participate in all procedures of the study. Exclusion criteria were (a) severe comorbidity as judged by one of the physicians of the study and (b) an inability to take part in all moments of the study. One participant originally included in the TIE cohort was retrospectively excluded from the present work due to the discovery of a severe comorbidity, a lymphatic leucaemia with extreme values of WBC and lymphocytes (133.2 and 130.0 × 10^9^ cells/L) that skewed the results.

### 2.2. Data Collection

At the baseline visit, a minimum elapsed time of four weeks since the last AECOPD was required; otherwise, the visit was postponed. The four-week delay was deemed a good balance between including patients possibly not fully recovered from an AECOPD and excluding patients suffering frequent AECOPDs. Participants underwent several investigations performed by trained research staff, including spirometry, blood samples, anthropometric measures, and questionnaires on smoking, drug use, comorbidities, and symptoms. 

Participants were followed up via a review of medical records from both primary and secondary care for up to three years (the end of the study) or until death.

### 2.3. Variables

#### 2.3.1. Outcome—Exacerbations

The outcome of this study was AECOPD frequency, defined as the annualised rate of AECOPDs during follow-up (that is, the total number of AECOPDs divided by the time of follow-up), grouped into four categories: (A) no AECOPD during follow-up; (B) > 0 but < 1 AECOPD/year; (C) ≥ 1 but < 2 AECOPDs/year; and (D) ≥ 2 AECOPDs/year. The number of AECOPDs was assessed by reviewing the participants’ medical records. The definition was an “unscheduled or scheduled health care visit with increased respiratory symptoms leading to inhalation of bronchodilators (at the health care facility), and/or treatment with oral corticosteroids, and/or treatment with antibiotics, and/or referral to emergency department, and/or hospitalisation due to COPD”. Events occurring within 14 days were regarded as the same AECOPD [18,19].

#### 2.3.2. Exacerbation History

AECOPDs in the year before the baseline visit were assessed by reviewing medical records, employing the same definition as above.

#### 2.3.3. Smoking

Current smoking was defined as self-reported daily or occasional smoking, i.e., the participant answering yes to whether they smoke daily or whether they smoke now and then. 

#### 2.3.4. Anthropometrics

Participants’ height and weight were registered. Body mass index (BMI) was calculated as weight (kg) divided by height (m) squared. 

#### 2.3.5. Symptoms

COPD-related symptoms were assessed with the modified Medical Research Council dyspnoea scale, ranging from 0 to 4, and the COPD Assessment Test (CAT), ranging from 0 to 40 [20]. For analytical purposes, the CAT results were treated as ratio scale data and analysed as continuous variables.

#### 2.3.6. Spirometry, Lung Function

Spirometry was performed fifteen minutes after the administration of 400 µg salbutamol [17], and FEV_1_, SVC, and FVC were registered. Swedish reference values were used to express FEV_1_ and FVC as per cent predicted [21,22]. Based on the FEV_1_ per cent predicted, participants were categorised into COPD grades 1–4 in line with The Global Initiative for Chronic Obstructive Lung Disease (GOLD) [1].

#### 2.3.7. Inhalational Drug Use

Current use of ICS, inhaled long-acting muscarinic antagonists (LAMA), or inhaled long-acting beta-2-agonists (LABA) was assessed with questionnaires, and defined as daily or intermittent use in the six months before the baseline visit.

#### 2.3.8. Comorbidities

The concurrence of asthma and/or heart failure was defined as present if the participants at the baseline visit answered yes in the questionnaire to whether they had or previously had had the disease of interest.

#### 2.3.9. Biomarkers of Systemic Inflammation

Venous blood samples were obtained at baseline for the analysis of all the biomarkers. The analyses were run at each study centre’s local hospital laboratory using clinical routine equipment (Appendix A). The biomarkers were analysed both as continuous variables and as dichotomised variables. 

For CRP, the Uppsala University Hospital laboratory’s upper reference range was used as the threshold (≥5 mg/L) for dichotomisation. For fibrinogen, the threshold suggested by Mannino et al. [12] (≥3.5 g/L) was used. The following blood cells were counted: platelets, WBC, neutrophils, lymphocytes, and monocytes. For the dichotomised analyses, the upper limit of the reference interval of the local laboratory at the Uppsala University Hospital laboratory (Table 1) was used for all blood cells besides lymphocytes, which were dichotomised based on the threshold value ≥ 1.8 × 10^9^ cells/L proposed by Semenzato et al. [23]. The haematological indices of interest for this study were calculated (PLR = platelets/lymphocytes; SII = platelets × neutrophils/lymphocytes; SIRI = monocytes × neutrophils/lymphocytes; and AISI = platelets × monocytes × neutrophils/lymphocytes) and analysed as both continuous and dichotomised variables. Since no established threshold values exist, the upper quartile defined high levels: PLR ≥ 169.1, SII ≥ 856, SIRI ≥ 2.024, and AISI ≥ 533.7. In the following, the dichotomous variable is denoted by the subscript “High” (e.g., WBC_High_, PLR_High_), indicating that the higher value is compared with the lower.

### 2.4. Statistical Analysis

Stata Statistical Software: Release 14.2 (StataCorp LP 2015, College Station, TX, USA) was used for managing data and statistical analyses. The significance level was set to 0.05. No corrections were made for multiple hypothesis testing.

Descriptive statistics were presented as number (n) and percentage (discrete data), mean ± standard deviation (SD, normally distributed continuous data) or median and interquartile range (IQR, non-normally distributed continuous data), as appropriate. Only a few participants had missing data (Supplementary Table S1), and there was no obvious pattern of missing items, so it was assumed that data were missing completely at random. Participants with missing data on a variable of interest were excluded from that specific analysis. Correlation analyses were performed using the Pearson correlation coefficient or Spearman’s rank correlation coefficient, and the variation inflation factor was employed to assess multicollinearity.

Ordinal logistic regression models were fitted with the four AECOPD frequency groups as outcomes. First, each variable of interest was analysed individually in bivariable models. Then, multivariable ordinal logistic models were fitted. Based on the previous literature, the variables with the strongest influence on AECOPD risk were deemed to be AECOPD history the year before baseline, age, sex, current smoking, BMI, CAT score, FEV_1_, and current ICS use. Therefore, they were all included as predictors [7,24]. Finally, the variables of focus for this study (biomarkers of systemic inflammation) were analysed one by one in a multivariable model adjusted by the predictors mentioned above. The proportional hazards (PH) assumption was assessed using the Brant test [25], which discovered that CRP as a continuous variable violated the assumption; therefore, it must be interpreted with caution. However, CRP analysed as a dichotomous variable did not violate the PH assumption. To test if adding the biomarker improved the model’s fit compared with using the adjustors only, likelihood ratio tests were performed (restricted to participants with no missing data on the biomarker of interest). 

All systemic inflammatory biomarkers of interest were analysed for interaction with exacerbation history (yes versus no) and FEV_1_ (< 50% versus ≥ 50% predicted) by adding interaction terms into the multivariable ordinal logistic models. Stratified analyses were performed for participants with and without a history of at least one AECOPD the year before baseline, with and without current use of ICS, and with FEV_1_ ≥ 50% predicted and < 50% predicted. Due to the smaller subgroups in the interaction analyses and smaller samples in the stratified analyses, the Brant test could not be used in all cases. 

Sensitivity analyses were performed. All participants with self-reported comorbid asthma were excluded from the study, and the multivariable analyses were repeated. Moreover, the multivariable analyses of CRP_High_, fibrinogen_High_, and WBC_High_ were repeated with the 10th decile of the variable of interest (i.e., the 10% with the highest values) excluded.

## 3. Results

In total, 571 participants were included. The cohort was followed for 1643 person years (mean time of follow-up: 2.9 years; range: 0.13–3 years). There was no loss to follow-up. Baseline data are presented in Table 1. Compared with participants with no AECOPD history, those with a history of at least one AECOPD the year before baseline had lower FEV_1_, used inhalational drugs more often, and had higher levels of blood-based biomarkers of systemic inflammation. Participants with FEV_1_ < 50% predicted (n = 200, 35%) had a higher degree of a history of AECOPD, used inhalational drugs more often, and had higher levels of inflammatory biomarkers than those with FEV_1_ ≥ 50% predicted (n = 371, 65%) (Supplementary Table S1).

Two hundred and sixty-three participants (46%) had ≥ 1 AECOPD (range 1–28, median 2) during follow-up (Table 2). The AECOPD frequency was (mean ± SD) 0.63 ± 1.2 AECOPDs/year (range 0–9.4). The proportion of frequent exacerbators, i.e., participants with ≥2 AECOPDs/year, was 10%. There were 54 deaths (9%) during follow-up.

### 3.1. Bivariable Analyses

No multicollinearity was discovered between variables, except for the haematological ratios. In the bivariable ordinal logistic regression models, most inflammatory biomarkers were associated with higher AECOPD frequency, including CRP_High_, fibrinogen, WBC, PLR, SII, SIRI, and AISI (Table 3).

### 3.2. Multivariable Analyses

Multivariable ordinal logistic regression models adjusted for AECOPD history the year before baseline, age, sex, current smoking, BMI, CAT score, FEV_1_, and current ICS use were fitted (Table 4). Only CRP_High_, fibrinogen_High_, and WBC_High_ were associated with higher AECOPD frequency in these models. In all cases, the likelihood ratio test indicated that adding the biomarker increased the model’s prognostic performance (*p* = 0.021, *p* = 0.019, and *p* = 0.016, respectively).

### 3.3. Interactions and Stratified Analyses

Neither AECOPD history nor FEV_1_ interacted with any of the included variables. Analyses were stratified by the current use of ICS at baseline (no/yes) (Supplementary Table S2), and among those currently using ICS, the results were similar to those of the main analysis except that monocytes_High_ was also associated with higher AECOPD frequency. No biomarker was associated with higher AECOPD frequency among participants not using ICS at baseline. Stratification for exacerbation history (no/yes) resulted in CRP_High_, fibrinogen_High_, and WBC_High_ being associated with higher AECOPD frequency only among participants with no history of AECOPD (Supplementary Table S3). Stratification for FEV_1_ ≥ 50 versus < 50% predicted resulted in CRP_High_, fibrinogen_High_, and WBC_High_ being associated with higher AECOPD frequency only among participants with FEV_1_ < 50% (Supplementary Table S4).

### 3.4. Sensitivity Analyses

When only participants without self-reported asthma were included (n = 378), CRP_High_, fibrinogen_High_, and WBC_High_ remained associated with higher AECOPD frequency. Additionally, WBC and monocytes_High_ were associated with AECOPDs (Supplementary Table S5).

Repeating the analyses with the 10th decile of each biomarker removed resulted in the following aORs (95% CI): CRP_High_ 2.01 (1.20–3.35), fibrinogen_High_ 1.45 (0.99–2.13), and WBC_High_ 2.01 (1.21–3.34).

## 4. Discussion

Our main finding was that after adjustment for clinically relevant confounders, high levels of CRP, fibrinogen, and WBC measured during stable-phase COPD were associated with a higher frequency of AECOPDs during a three-year follow-up period. This indicates that incorporating these biomarkers could enhance the performance of predictive models. PLR, SII, SIRI, and AISI, on the other hand, were related to higher AECOPD frequency in the unadjusted analyses only, which indicates limited clinical value.

We found in the adjusted analysis that participants with a CRP ≥ 5 mg/L had an approximately 1.6 times higher risk for higher AECOPD frequency than those with lower CRP. To the best of our knowledge, ours is the first original research paper to show that CRP in a stable phase independently predicts any future AECOPD frequency. Previous studies have found CRP to be predictive of AECOPDs, but only in unadjusted analyses [6,26,27], in combination with other biomarkers [10,11], or only regarding AECOPDs leading to hospitalisation [28,29,30]. The first to report an association between CRP and future AECOPDs were Dahl et al. [28]. That association was further explored by Hurst et al. in the landmark ECLIPSE (The Evaluation of COPD Longitudinally to Identify Predictive Surrogate End-Points) study, where participants were assessed during a stable phase of COPD and followed for three years [6]. Agusti et al. used the ECLIPSE data to study persistently elevated inflammatory biomarkers (WBC, CRP, interleukin-6, and fibrinogen) and found that participants with ≥2 persistently elevated biomarkers have a higher annual rate of AECOPDs than those without [10]. A similar approach was employed by Thomsen et al. to demonstrate that the number of elevated biomarkers (WBC, CRP, and fibrinogen) at baseline predicts AECOPDs [11]. In 2019, a meta-analysis utilising data from four papers (including the work by Hurst et al. mentioned above) found that higher levels of stable-phase CRP are associated with AECOPDs, but it is not clearly stated if adjustment for confounders was made [31]. A probable explanation for the inconsistent results is whether or not CRP was analysed as a continuous variable. We found that CRP, analysed as a continuous variable, is a poor predictor of AECOPD, likely due to a nonlinear risk increase. The three studies reporting associations only in the unadjusted analyses analysed CRP continuously [6,26,27]. The studies reporting associations between CRP in combination with other biomarkers and AECOPDs used dichotomous CRP [10,11]. The studies that found associations only with AECOPD hospitalisations used dichotomous or categorised CRP [28,29,30]. The first two of these studies did not have non-severe AECOPDs as an outcome [28,29], and the third restricted its analyses to people with FEV_1_ ≥ 50% and ≤ 70% predicted [30]. Our stratified analyses showed that the prognostic value of CRP regarding future AECOPDs is better among participants with FEV_1_ < 50% predicted, probably due to the larger degree of systemic inflammation and higher risk of AECOPD in this group.

Fibrinogen ≥ 3.5 g/L was related to a higher AECOPD frequency in the adjusted analysis, similar to previous reports using the same cut-off [12,32,33]. Also, when analysed dichotomously in combination with other biomarkers, fibrinogen is prognostic of AECOPDs [10,11,30,34]. Some studies using continuous fibrinogen have reported an association with future AECOPDs [35] or severe AECOPDs [12,36], but others found such an association only in unadjusted analyses [6,27]. The meta-analysis by Fermont et al. showed that fibrinogen may predict AECOPDs, but it is unclear if there was any adjustment for confounders [31]. We infer that fibrinogen ≥ 3.5 g/L, measured in stable-phase COPD, is prognostic of future AECOPDs. Future research should investigate if fibrinogen could be a part of a composite AECOPD prediction tool.

Concerning WBC, we found that levels > 9 × 10^9^ cells/L were associated with higher AECOPD frequency after adjustment for confounders, whereas the continuous WBC was not. In their report from ECLIPSE, Hurst and co-workers reported an association between continuous WBC and more AECOPDs after adjustment for confounders [6]. In contrast, others have reported WBC to be associated with AECOPDs in the unadjusted analysis only [26,37]. In their meta-analysis of three papers, Fermont et al. concluded that stable-phase WBC is not associated with AECOPDs, but again, it is not clear if their estimates were adjusted for confounders [31]. As mentioned above, WBC was included in the works by Agusti et al. and Thomsen et al. and was found to be predictive of AECOPDs when analysed in combination with other biomarkers, and in these studies WBC was dichotomised [10,11]. There is reason to believe that WBC, similar to CRP and fibrinogen, is of limited use when analysed as a continuous variable, probably owing to a nonlinear risk increase. However, evidence, including that in our present work, suggests that dichotomised WBC is a promising biomarker. Future research is needed to establish the value of WBC and to find the optimal threshold. 

There was no association between any of the blood cell indices (PLR, SII, SIRI, and AISI) and AECOPD frequency in the adjusted analyses, although associations were found in the bivariable analyses. PLR is higher in stable COPD compared with healthy controls. PLR is also higher in AECOPD compared with stable COPD [38]. SII, SIRI and AISI are novel biomarkers, with only a few reported publications in COPD [14,15,39]. SII integrates neutrophils, platelets, and lymphocytes, SIRI integrates neutrophils, monocytes, and lymphocytes, and AISI integrates all four: neutrophils, monocytes, platelets, and lymphocytes. In a cohort study of patients previously hospitalised due to AECOPD, Liu et al. reported that stable-phase PLR, SII, and SIRI predict AECOPDs after adjustment for confounders [14]. However, they did not adjust for AECOPD history, which is probably why our results could not confirm theirs. It is probable that the indices are merely markers of previous AECOPDs—the strongest risk factor of future AECOPDs [7]. This was the first study on the AISI and the second on the PLR, SII, and SIRI concerning future AECOPDs, but our results are discouraging. The blood cell indices seem to have no independent prognostic value.

The available evidence, including the present work, indicates that several biomarkers of systemic inflammation have potential as prognostic factors of AECOPD. Although single biomarkers often show weak associations with outcomes, contribute less to the predictions than clinical data, and show heterogeneity across studies [27,40], blood-based biomarkers may prove useful since they are objective and likely less prone to errors than information gathered during a health care professional’s interview with a patient. Moreover, several studies show the additive (independent) value of biomarkers, as with high levels of WBC, fibrinogen, and CRP in the present analysis, indicating that they could be included in composite risk scoring systems [14,41]. Future research should aim to integrate inflammatory biomarkers in, for instance, statistical risk prediction tools [42]. There might be several explanations for the association between biomarkers and AECOPD observed in this study. Systemic inflammation as measured by the biomarkers studied here may be a feature of more severe COPD, carrying a higher AECOPD risk. It may also be an effect of comorbidities that are associated with both inflammation and an increased AECOPD risk, such as diabetes or heart diseases [7]. Future research, including genome-wide association studies, may shed more light on the pathways leading to systemic inflammation in COPD.

The main strengths of the present study were the thorough and complete follow-up, few missing data, and long follow-up time of up to three years. The COPD diagnosis was confirmed via spirometry, and there was rigorous testing of various parameters at the baseline visit, enabling adjustment for most of the relevant confounders. Moreover, assessing AECOPDs in medical records ensured that only clinically relevant events were recorded and eliminates recall bias. The results are likely generalisable to a North European population since participants were recruited in three different Swedish regions and from both primary and secondary care.

### Limitations

Due to its observational nature, no conclusion on causality can be drawn from the present study. The use of medical records as the source for our outcome data may have led to missed AECOPDs in the case that a participant suffered an event when travelling in other regions or if there were participants self-treating their AECOPDs (e.g., having been prescribed oral corticosteroids and/or antibiotics in advance, to use in the eventuality of an AECOPD). Furthermore, all data on current drug use and comorbidities were self-reported, which means that misunderstandings or misconceptions may have created flaws in them. There were also no data on some known risk factors of AECOPD, including bronchiectasis and gastro-oesophageal reflux disease, precluding adjustment for those. Moreover, only 8% of the study population had very severe COPD (GOLD grade 4), so the present results may not be generalisable to that group. Measuring a biomarker at a single time point—as in this study—may not be representative of that individual’s “true” value, depending on the variability in and reliability of the specific biomarker. In the case of inflammatory biomarkers, a single measurement also makes the data vulnerable to residual inflammation after inflammatory conditions. At the study visit, however, participants were required to feel healthy, and if at least four weeks had not passed since the last AECOPD, the visit was postponed. Finally, in this study, each of the three study centres had its own laboratories with, in part, different equipment, which may have introduced bias. However, all laboratories were certified hospital laboratories, and the analyses were part of their standard range. 

## 5. Conclusions

CRP, fibrinogen, and WBCs measured during stable-phase COPD are independently associated with AECOPDs and improve AECOPD prediction. A thorough clinical risk evaluation remains critical in managing patients with COPD, but CRP, fibrinogen and WBC may be valuable as part of a composite AECOPD risk assessment tool. In contrast, the PLR, SII, SIRI, and AISI are of no additional value to clinical risk stratification. 

## Figures and Tables

**Table 1:** Baseline characteristics of the study population; mean ± SD, n (%), or median (IQR).

	Total Cohort	≥ 1 AECOPD the Year before Baseline	
	n = 571	No, n = 405 (71%)	Yes, n = 166 (29%)
Age	69 ± 8	69 ± 8	69 ± 7
Female sex	334 (58%)	226 (56%)	108 (65%)
Smoking history			
Current smoker	166 (29%)	115 (29%)	51 (31%)
Ex-smoker	394 (69%)	283 (70%)	111 (67%)
Never-smoker	9 (2%)	5 (1%)	4 (2%)
BMI, kg/m^2^	26 (23–30)	27 (23–30)	26 (23–30)
CAT score	13 ± 7	12 ± 7	15 ± 9
CAT ≥ 10	354 (62%)	238 (59%)	116 (70%)
mMRC score	1 (1–3)	1 (1–2)	2 (1–3)
mMRC ≥ 2	253 (44%)	152 (38%)	101 (61%)
FEV_1_, % ^†^	57 ± 18	59 ± 17	50 ± 18
GOLD grade			
1 (FEV_1_ ≥ 80% ^†^)	57 (10%)	48 (12%)	9 (5%)
2 (50% ^†^ ≤ FEV_1_ < 80% ^†^)	314 (55%)	234 (58%)	80 (48%)
3 (30% ^†^ ≤ FEV_1_ < 50% ^†^)	155 (27%)	103 (25%)	52 (31%)
4 (FEV_1_ < 30% †)	45 (8%)	20 (5%)	25 (15%)
ICS, current use	388 (70%)	244 (62%)	144 (89%)
LABA, current use	364 (65%)	229 (57%)	135 (83%)
LAMA, current use	402 (71%)	265 (66%)	137 (84%)
Asthma	190 (34%)	124 (31%)	66 (40%)
Heart failure	29 (5%)	14 (3%)	15 (9%)
CRP, mg/L	2.5 (1.2–4.9)	2.4 (1.2–4.7)	2.8 (1.1–5.8)
CRP ≥ 5 mg/L	136 (24%)	87 (22%)	49 (30%)
Fibrinogen, g/L	3.6 (3.1–4.0)	3.5 (3.0–3.9)	3.7 (3.3–4.3)
Fibrinogen ≥ 3.5 g/L	301 (55%)	197 (52%)	104 (63%)
WBC, ×10^9^ cells/L	7.6 (6.5–9.0)	7.4 (6.4–8.7)	8.3 (7.1–9.5)
WBC > 9 ×10^9^ cells/L	137 (24%)	83 (21%)	54 (33%)
Platelets, ×10^9^ cells/L	267 ± 69	265 ± 66	274 ± 74
Platelets > 350 × 10^9^ cells/L	60 (11%)	36 (9%)	24 (15%)
Neutrophils, ×10^9^ cells/L	4.7 (3.7–5.7)	4.5 (3.5–5.4)	5.2 (4.1–6.3)
Neutrophils > 5.4 × 10^9^ cells/L	167 (30%)	98 (25%)	69 (42%)
Lymphocytes, ×10^9^ cells/L	2.0 (1.6–2.4)	2.0 (1.6–2.5)	2.0 (1.5–2.4)
Lymphocytes ≥ 1.8 × 10^9^ cells/L	359 (64%)	257 (65%)	102 (62%)
Monocytes, ×10^9^ cells/L	0.6 (0.5–0.7)	0.6 (0.5–0.7)	0.6 (0.5–0.8)
Monocytes > 0.8 ×10^9^ cells/L	85 (15%)	53 (13%)	32 (20%)
PLR	131 (103–169)	129 (103–168)	138 (108–172)
PLR ≥ 169.1	135 (25%)	93 (24%)	42 (26%)
SII	596 (420–856)	559 (405–777)	708 (485–974)
SII ≥ 856	136 (25%)	76 (20%)	60 (38%)
SIRI	1.4 (1.0–2.0)	1.3 (0.9–1.8)	1.8 (1.2–2.3)
SIRI ≥ 2.024	140 (25%)	74 (19%)	66 (40%)
AISI	362 (245–534)	336 (220–479)	472 (283–676)
AISI ≥ 533.7	135 (25%)	72 (19%)	63 (39%)

^†^ of predicted. Note: AECOPD, acute exacerbation of COPD; BMI, body mass index; CAT, COPD Assessment Test; mMRC, modified Medical Research Council dyspnoea scale; FEV_1_, forced expiratory volume in one second; GOLD, The Global Initiative for Obstructive Lung Disease; ICS, inhaled corticosteroids; LABA, inhaled long-acting beta-2-agonists; LAMA, inhaled long-acting muscarinic antagonists; CRP, C-reactive protein; WBC, white blood cell count; PLR, platelet-to-lymphocyte ratio; SII, systemic immune inflammation index; SIRI, systemic inflammation response index; AISI, aggregate index of systemic inflammation.

**Table 2:** Frequency of AECOPD during follow-up (n = 571); n (%).

AECOPDs/Years	Total Cohort	≥ 1 AECOPD the Year before Baseline	
	n = 571	No, n = 405	Yes, n = 166
None	309 (54%)	265 (65%)	44 (27%)
> 0, < 1	138 (24%)	92 (23%)	46 (28%)
≥ 1, < 2	67 (12%)	33 (8%)	34 (20%)
≥ 2	57 (10%)	15 (4%)	42 (25%)

Note: AECOPD, acute exacerbation of COPD.

**Table 3:** Association between blood-based inflammatory biomarkers and future AECOPD frequency. Unadjusted ordinal logistic regression models. Each odds ratio represents a separate model.

Biomarker	n	OR	95% CI
CRP, per 1 mg/L	565	1.02	0.99–1.04
CRP ≥ 5 mg/L	565	1.86	1.29–2.67
Fibrinogen, per 1 g/L	545	1.59	1.26–2.00
Fibrinogen ≥ 3.5 g/L	545	2.01	1.45–2.79
WBC, per 1 ×10^9^ cells/L	568	1.16	1.08–1.24
WBC > 9 ×10^9^ cells/L	568	2.18	1.52–3.13
Platelets, per 100 ×10^9^ cells/L	547	1.36	1.07–1.71
Platelets > 350 ×10^9^ cells/L	547	1.42	0.86–2.37
Neutrophils, per 1 × 10^9^ cells/L	564	1.23	1.12–1.36
Neutrophils > 5.4 ×10^9^ cells/L	564	1.92	1.36–2.71
Lymphocytes, per 1 × 10^9^ cells/L	562	0.90	0.73–1.11
Lymphocytes ≥ 1.8 × 10^9^ cells/L	562	0.96	0.69–1.33
Monocytes, per 0.1 × 10^9^ cells/L	562	1.10	1.03–1.18
Monocytes > 0.8 × 10^9^ cells/L	562	1.81	1.17–2.81
PLR, per 100 units	541	1.32	1.02–1.71
PLR ≥ 169.1	541	1.20	0.83–1.73
SII, per 100 units	541	1.08	1.04–1.12
SII ≥ 856	541	1.52	1.05–2.19
SIRI, per 1 unit	562	1.28	1.12–1.47
SIRI ≥ 2.024	562	1.76	1.23–2.52
AISI, per 100 units	541	1.09	1.04–1.14
AISI ≥ 533.7	541	2.03	1.40–2.92

Note: OR, odds ratio; CI, confidence interval; CRP, C-reactive protein; WBC, white blood cell count; PLR, platelet-to-lymphocyte ratio; SII, systemic immune inflammation index; SIRI, systemic inflammation response index; AISI, aggregate index of systemic inflammation.

**Table 4:** Association between blood-based inflammatory biomarkers and future AECOPD frequency. Ordinal logistic regression models adjusted for AECOPD history the year before baseline, age, sex, current smoking, BMI, CAT score, FEV_1_, and current ICS use. Each adjusted odds ratio represents a separate model.

Biomarker	n	aOR	95% CI
CRP, per 1 mg/L	548	1.00	0.97–1.03
CRP ≥ 5 mg/L	548	1.64	1.08–2.49
Fibrinogen, per 1 g/L	528	1.13	0.87–1.47
Fibrinogen ≥ 3.5 g/L	528	1.55	1.07–2.24
WBC, per 1 × 10^9^ cells/L	551	1.07	0.99–1.15
WBC > 9 × 10^9^ cells/L	551	1.65	1.10–2.47
Platelets, per 100 × 10^9^ cells/L	530	1.26	0.97–1.64
Platelets > 350 × 10^9^ cells/L	530	1.17	0.67–2.03
Neutrophils, per 1 × 10^9^ cells/L	547	1.03	0.92–1.15
Neutrophils > 5.4 × 10^9^ cells/L	547	1.17	0.79–1.72
Lymphocytes, per 1 × 10^9^ cells/L	545	1.00	0.78–1.26
Lymphocytes ≥ 1.8 × 10^9^ cells/L	545	1.15	0.80–1.67
Monocytes, per 0.1 × 10^9^ cells/L	545	1.03	0.95–1.11
Monocytes > 0.8 × 10^9^ cells/L	545	1.59	0.99–2.56
PLR, per 100 units	524	1.10	0.82–1.46
PLR ≥ 169.1	524	0.88	0.58–1.33
SII, per 100 units	524	1.03	0.99–1.07
SII ≥ 856	524	0.81	0.53–1.23
SIRI	545	1.05	0.90–1.22
SIRI ≥ 2.024	545	0.80	0.53–1.21
AISI, per 100 units	524	1.03	0.99–1.07
AISI ≥ 533	524	1.01	0.66–1.53

Notes: aOR, adjusted odds ratio; CI, confidence interval; CRP, C-reactive protein; WBC, white blood cell count; PLR, platelet-to-lymphocyte ratio; SII, systemic immune inflammation index; SIRI, systemic inflammation response index; AISI, aggregate index of systemic inflammation.

## Data Availability

Swedish personal data protection legislation prohibits the free sharing of study data. However, upon reasonable request and after approval by the Swedish Ethical Review Authority, anonymised patient-level data can be made available by the corresponding author.

## Supplementary Materials

The following are available online at https://www.mdpi.com/article/10.3390/jcm13133855/s1, Table S1: Missing data and baseline characteristics of the study population stratified by FEV_1_, Table S2: Analyses stratified by current use of inhaled corticosteroids at baseline, Table S3: Analyses stratified by AECOPD history, Table S4: Analyses stratified by FEV_1_ ≥ 50 versus < 50% predicted, Table S5: Sensitivity analysis, including only participants without asthma.

## Appendix A. Details on Laboratory Equipment

Blood cells were analysed with Cell-Dyn 4000 and Sapphire (Abbott Laboratories, Abbott Park, IL, USA) and Sysmex XN-10 and XN-20 (Sysmex Corporation, Kobe, Japan).

CRP was analysed with Architect ci8200 and c16000 (Abbott Laboratories, IL, USA), Cobas 6000 (Roche Group, Basel, Switzerland), and ADVIA 1800 (Siemens Healthcare GmbH, Erlangen, Germany).

Plasma fibrinogen was analysed with Architect c16000, Cobas 6000, STA R Max (Stago Group, Asnières sur Seine Cedex, France), and Sysmex CS2100i (Sysmex Corporation, Kobe, Japan).
