# Supplementary material for: CRP, Fibrinogen, White Blood Cells, and Blood Cell Indices as Prognostic Biomarkers of Future COPD Exacerbation Frequency: The TIE Cohort Study
Source: J Clin Med. 2024 Jun 30;13(13):3855. doi: 10.3390/jcm13133855 (PMC11242174; doi:10.3390/jcm13133855)
Supplement: Supplementary file 1 [file jcm-13-03855-s001.zip › jcm-3056575-supplementary.pdf]

Supplementary material

CRP, fibrinogen, white blood cells, and blood cell indices as prognostic biomarkers of future COPD exacerbation frequency: the TIE cohort study

Ellingsen, J., Janson, C., Bröms, K., Hårdstedt, M., Högman, M., Lisspers, K., Palm, A., Ställberg, B., Malinovschi, A.

Contents

Table S1. Missing data and baseline characteristics of the study population stratified by FEV_1 3

Table S2. Analyses stratified by current use of inhaled corticosteroids at baseline 4

Table S3. Analyses stratified by AECOPD history 5

Table S4. Analyses stratified by $FEV_1 \geq 50$ versus < 50% predicted 6

Table S5. Sensitivity analysis, including only participants without asthma 7

Table S1. Missing data and baseline characteristics of the study population stratified by FEV_1. Mean ± SD, n (%), or median (IQR).

	Missing data	FEV_1 at baseline	
		**≥ 50%†**, n = 371 (65%)	**< 50%†**, n = 200 (35%)
Age	0	68±8	69±8
Female sex	0	215 (58%)	119 (60%)
Smoking history	2		
Current smoker		114 (31%)	52 (26%)
Ex-smoker		251 (68%)	143 (72%)
Never-smoker		4 (1%)	5 (3%)
BMI, kg/m^2	3	27 (24–30)	25 (22–30)
CAT score	0	11 ± 7	15 ± 8
FEV_1, %†	0	67 ± 12	37 ± 9
≥ 1 AECOPD the year before baseline	0	89 (24%)	77 (39%)
ICS, current use	13	219 (61%)	169 (86%)
LABA, current use	8	199 (55%)	165 (83%)
LAMA, current use	7	229 (63%)	173 (87%)
Asthma	4	117 (32%)	73 (37%)
Heart failure	0	22 (6%)	7 (4%)
CRP, mg/L	6	2.4 (1.1–4.4)	2.6 (1.3–5.9)
CRP ≥ 5 mg/L	6	75 (20%)	61 (31%)
Fibrinogen, g/L	26	3.4 (3.0–3.9)	3.7 (3.3–4.3)
Fibrinogen ≥ 3.5 g/L	26	175 (49%)	126 (66%)
WBC, ×10^9 cells/L	3	7.4 (6.4–8.7)	8.0 (6.7–9.4)
WBC > 9 ×10^9 cells/L	3	74 (20%)	63 (32%)
Platelets, ×10^9 cells/L	24	265 ± 65	273 ± 75
Platelets > 350 ×10^9 cells/L	24	37 (10%)	23 (12%)
Neutrophils, ×10^9 cells/L	7	4.5 (3.6–5.4)	5.1 (4.0–6.1)
Neutrophils > 5.4 ×10^9 cells/L	7	90 (25%)	77 (39%)
Lymphocytes, ×10^9 cells/L	9	2.0 (1.6–2.5)	1.9 (1.5–2.4)
Lymphocytes ≥ 1.8 ×10^9 cells/L	9	236 (64%)	123 (63%)
Monocytes, ×10^9 cells/L	9	0.6 (0.5–0.7)	0.6 (0.5–0.8)
Monocytes > 0.8 ×10^9 cells/L	9	43 (12%)	42 (21%)
PLR	30	128 (98–164)	141 (110–180)
PLR ≥ 169.1	30	79 (22%)	56 (30%)
SII	9	560 (405–787)	656 (478–1039)
SII ≥ 856	9	71 (20%)	65 (34%)
SIRI	30	1.3 (1.0–1.8)	1.7 (1.1–2.4)
SIRI ≥ 2.024	30	67 (18%)	73 (37%)
AISI	30	337 (228–478)	442 (280–710)
AISI ≥ 533.7	30	64 (18%)	71 (38%)

Note: † of predicted. AECOPD, acute exacerbation of chronic obstructive pulmonary disease; BMI, body mass index; CAT, COPD Assessment Test; FEV_1, forced expiratory volume in one second; GOLD, The Global initiative for Obstructive Lung Disease; ICS, inhaled corticosteroids; LABA, inhaled long-acting beta-2-agonists; LAMA, inhaled long-acting muscarinic antagonists; CRP, C-reactive protein; WBC, white blood cell count; PLR, platelet-to-lymphocyte ratio; SII, systemic immune-inflammation index; SIRI, systemic inflammation response index; AISI, aggregate index of systemic inflammation.

Table S2. Analyses stratified by current use of inhaled corticosteroids at baseline.

Association between blood-based inflammatory biomarkers and future AECOPD frequency stratified by current use of inhaled corticosteroids at baseline. Ordinal logistic regression models adjusted for AECOPD history the year before baseline, age, sex, current smoking, BMI, CAT score, FEV_1, and current ICS use. Each adjusted odds ratio represents a separate model.

	Current use of ICS at baseline					
	No			Yes		
**Biomarker**	**n**	**aOR**	**95% CI**	**n**	**aOR**	**95% CI**
CRP, per 1 mg/L	167	0.90	0.80–1.004	381	1.01	0.98–1.04
CRP ≥ 5 mg/L	167	0.67	0.26–1.74	381	**2.04**	**1.26–3.30**
Fibrinogen, per 1 g/L	163	0.65	0.37–1.13	365	1.31	0.97–1.77
Fibrinogen ≥ 3.5 g/L	163	0.80	0.37–1.76	365	**1.79**	**1.17–2.74**
WBC, per 1 $\times 10^9$ cells/L	169	1.02	0.84–1.24	382	1.07	0.99–1.16
WBC > 9 $\times 10^9$ cells/L	169	1.06	0.37–3.01	382	**1.76**	**1.13–2.75**
Platelets, per 100 $\times 10^9$ cells/L	167	1.07	0.61–1.89	363	1.34	0.99–1.82
Platelets > 350 $\times 10^9$ cells/L	167	0.69	0.17–2.79	363	1.40	0.75–2.62
Neutrophils, per 1 $\times 10^9$ cells/L	167	1.11	0.88–1.40	380	1.00	0.88–1.14
Neutrophils > 5.4 $\times 10^9$ cells/L	167	1.26	0.51–3.15	380	1.19	0.77–1.84
Lymphocytes, per 1 $\times 10^9$ cells/L	167	0.66	0.39–1.12	378	1.10	0.84–1.46
Lymphocytes ≥ 1.8 $\times 10^9$ cells/L	167	1.33	0.59–3.00	378	1.07	0.71–1.63
Monocytes, per 0.1 $\times 10^9$ cells/L	167	1.01	0.86–1.19	378	1.03	0.94–1.12
Monocytes > 0.8 $\times 10^9$ cells/L	167	1.06	0.34–3.25	378	**1.72**	**1.002–2.94**
PLR, per 100 units	165	1.25	0.71–2.19	359	1.05	0.75–1.47
PLR ≥ 169.1	165	0.98	0.38–2.47	359	0.89	0.56–1.42
SII, per 100 units	165	1.04	0.97–1.12	359	1.02	0.97–1.07
SII ≥ 856	165	1.40	0.55–3.57	359	0.73	0.45–1.17
SIRI	167	1.18	0.93–1.49	378	0.95	0.79–1.16
SIRI ≥ 2.024	167	0.91	0.34–2.42	378	0.76	0.48–1.20
AISI, per 100 units	165	1.04	0.98–1.10	359	1.02	0.96–1.09
AISI ≥ 533	165	1.40	0.56–3.47	359	0.93	0.58–1.49

Note: Bold figures indicate statistical significance at the 0.05 level (1 is not included in the 95% CI).

AECOPD, acute exacerbation of chronic obstructive pulmonary disease; ICS, inhaled corticosteroids; LAMA, inhaled long-acting muscarinic antagonists; FEV_1, forced expiratory volume in one second; BMI, body mass index; CAT, COPD assessment test; aOR, adjusted odds ratio; CI, confidence interval; CRP, C-reactive protein; WBC, white blood cell count; PLR, platelet-to-lymphocyte ratio; SII, systemic immune-inflammation index; SIRI, systemic inflammation response index; AISI, aggregate index of systemic inflammation.

Table S3. Analyses stratified by AECOPD history.

Association between blood-based inflammatory biomarkers and future AECOPD frequency stratified by history of AECOPD the year before baseline. Ordinal logistic regression models adjusted for age, sex, current smoking, BMI, CAT score, FEV_1, and current ICS use. Each adjusted odds ratio represents a separate model.

	Number of AECOPD the year before baseline					
	None			≥1		
**Biomarker**	**n**	**aOR**	**95% CI**	**n**	**aOR**	**95% CI**
CRP, per 1 mg/L	386	0.99	0.95–1.03	162	1.01	0.96–1.05
CRP ≥ 5 mg/L	386	**1.80**	**1.07–3.03**	162	1.24	0.60–2.57
Fibrinogen, per 1 g/L	367	1.24	0.89–1.72	161	1.04	0.66–1.64
Fibrinogen ≥ 3.5 g/L	367	**1.67**	**1.04–2.69**	161	0.93	0.45–1.91
WBC, per 1 $\times 10^9$ cells/L	389	1.08	0.99–1.18	162	1.01	0.88–1.17
WBC > 9 $\times 10^9$ cells/L	389	**1.70**	**1.01–2.86**	162	1.42	0.76–2.64
Platelets, per 100 $\times 10^9$ cells/L	372	1.19	0.84–1.68	158	1.40	0.92–2.12
Platelets > 350 $\times 10^9$ cells/L	372	0.95	0.45–1.99	158	1.38	0.57–3.35
Neutrophils, per 1 $\times 10^9$ cells/L	387	1.05	0.91–1.22	160	0.97	0.81–1.16
Neutrophils > 5.4 $\times 10^9$ cells/L	387	1.17	0.71–1.94	160	1.01	0.54–1.89
Lymphocytes, per 1 $\times 10^9$ cells/L	385	0.99	0.74–1.32	160	1.02	0.66–1.56
Lymphocytes ≥ 1.8 $\times 10^9$ cells/L	385	1.17	0.73–1.86	160	1.16	0.63–2.15
Monocytes, per 0.1 $\times 10^9$ cells/L	385	1.06	0.96–1.17	160	0.97	0.85–1.10
Monocytes > 0.8 $\times 10^9$ cells/L	385	1.57	0.86–2.86	160	1.44	0.64–3.24
PLR, per 100 units	368	1.07	0.74–1.55	156	1.18	0.73–1.91
PLR ≥ 169.1	368	0.99	0.59–1.65	156	0.70	0.35–1.39
SII, per 100 units	368	1.02	0.97–1.07	156	1.04	0.97–1.11
SII ≥ 856	368	0.69	0.39–1.25	156	0.83	0.44–1.57
SIRI	385	1.15	0.96–1.37	160	0.83	0.63–1.08
SIRI ≥ 2.024	385	0.85	0.49–1.50	160	0.68	0.36–1.28
AISI, per 100 units	368	1.04	0.99–1.09	156	1.00	0.92–1.09
AISI ≥ 533	368	1.01	0.57–1.77	156	0.88	0.46–1.68

Note: Bold figures indicate statistical significance at the 0.05 level (1 is not included in the 95% CI). AECOPD, acute exacerbation of chronic obstructive pulmonary disease; ICS, inhaled corticosteroids; LAMA, inhaled long-acting muscarinic antagonists; FEV_1, forced expiratory volume in one second; BMI, body mass index; CAT, COPD assessment test; aOR, adjusted odds ratio; CI, confidence interval; CRP, C-reactive protein; WBC, white blood cell count; PLR, platelet-to-lymphocyte ratio; SII, systemic immune-inflammation index; SIRI, systemic inflammation response index; AISI, aggregate index of systemic inflammation.

Table S4. Analyses stratified by $FEV_1 \geq 50$ versus < 50% predicted.

Association between blood-based inflammatory biomarkers and future AECOPD frequency stratified by baseline $FEV_1 \geq 50$ versus < 50% predicted. Ordinal logistic regression models adjusted for AECOPD history the year before baseline, age, sex, current smoking, BMI, CAT score, FEV_1, and current ICS use. Each adjusted odds ratio represents a separate model.

	**$FEV_1 \geq 50$% predicted**			**$FEV_1 < 50$% predicted**		
**Biomarker**	**n**	**aOR**	**95% CI**	**n**	**aOR**	**95% CI**
CRP, per 1 mg/L	355	1.02	0.97–1.07	193	0.99	0.95–1.02
CRP ≥ 5 mg/L	355	1.35	0.76–2.41	193	**2.05**	**1.09–3.88**
Fibrinogen, per 1 g/L	342	1.02	0.71–1.46	186	1.29	0.86–1.93
Fibrinogen ≥ 3.5 g/L	342	1.13	0.69–1.82	186	**2.51**	**1.37–4.56**
WBC, per 1 $\times 10^9$ cells/L	357	1.05	0.93–1.19	194	1.09	0.98–1.20
WBC > 9 $\times 10^9$ cells/L	357	1.47	0.84–2.59	194	**1.89**	**1.03–3.47**
Platelets, per 100 $\times 10^9$ cells/L	343	1.15	0.79–1.67	187	1.39	0.95–2.05
Platelets > 350 $\times 10^9$ cells/L	343	0.93	0.42–2.05	187	1.42	0.63–3.22
Neutrophils, per 1 $\times 10^9$ cells/L	355	1.06	0.90–1.24	192	1.01	0.86–1.19
Neutrophils > 5.4 $\times 10^9$ cells/L	355	1.15	0.67–1.97	192	1.16	0.65–2.09
Lymphocytes, per 1 $\times 10^9$ cells/L	354	0.91	0.68–1.24	191	1.20	0.79–1.82
Lymphocytes ≥ 1.8 $\times 10^9$ cells/L	354	0.95	0.58–1.54	191	1.46	0.82–2.61
Monocytes, per 0.1 $\times 10^9$ cells/L	354	1.05	0.94–1.18	191	1.00	0.90–1.12
Monocytes > 0.8 $\times 10^9$ cells/L	354	1.20	0.59–2.43	191	1.89	0.96–3.70
PLR, per 100 units	340	1.09	0.73–1.62	184	1.09	0.72–1.65
PLR ≥ 169.1	340	1.07	0.62–1.87	184	0.71	0.38–1.33
SII, per 100 units	340	1.03	0.97–1.09	184	1.03	0.97–1.09
SII ≥ 856	340	0.71	0.38–1.29	184	0.91	0.50–1.68
SIRI	354	1.11	0.91–1.35	191	0.97	0.77–1.21
SIRI ≥ 2.024	354	0.57	0.31–1.06	191	0.99	0.55–1.78
AISI, per 100 units	340	1.03	0.98–1.09	184	1.03	0.96–1.10
AISI ≥ 533	340	1.02	0.56–1.86	184	0.98	0.54–1.76

Note: Bold figures indicate statistical significance at the 0.05 level (1 is not included in the 95% CI).

AECOPD, acute exacerbation of chronic obstructive pulmonary disease; ICS, inhaled corticosteroids; LAMA, inhaled long-acting muscarinic antagonists; FEV_1, forced expiratory volume in one second; BMI, body mass index; CAT, COPD assessment test; aOR, adjusted odds ratio; CI, confidence interval; CRP, C-reactive protein; WBC, white blood cell count; PLR, platelet-to-lymphocyte ratio; SII, systemic immune-inflammation index; SIRI, systemic inflammation response index; AISI, aggregate index of systemic inflammation.

Table S5. Sensitivity analysis, including only participants without asthma (n = 378).

Association between blood-based inflammatory biomarkers and future AECOPD frequency restricted to participants without comorbid asthma. Ordinal logistic regression models adjusted for AECOPD history the year before baseline, age, sex, current smoking, BMI, CAT score, FEV_1, and current ICS use. Each adjusted odds ratio represents a separate model.

Biomarker	n	aOR	95% CI
CRP, per 1 mg/L	363	1.01	0.97–1.04
CRP ≥ 5 mg/L	363	**1.82**	**1.07–3.08**
Fibrinogen, per 1 g/L	348	1.26	0.90–1.76
Fibrinogen ≥ 3.5 g/L	348	**1.77**	**1.10–2.83**
WBC, per 1 $\times 10^9$ cells/L	366	**1.12**	**1.02–1.23**
WBC > 9 $\times 10^9$ cells/L	366	**1.73**	**1.03–2.90**
Platelets, per 100 $\times 10^9$ cells/L	353	1.09	0.78–1.53
Platelets > 350 $\times 10^9$ cells/L	353	0.93	0.45–1.92
Neutrophils, per 1 $\times 10^9$ cells/L	363	1.10	0.96–1.27
Neutrophils > 5.4 $\times 10^9$ cells/L	363	1.18	0.73–1.93
Lymphocytes, per 1 $\times 10^9$ cells/L	361	1.05	0.77–1.44
Lymphocytes ≥ 1.8 $\times 10^9$ cells/L	361	1.29	0.81–2.06
Monocytes, per 0.1 $\times 10^9$ cells/L	361	1.07	0.97–1.17
Monocytes > 0.8 $\times 10^9$ cells/L	361	**1.89**	**1.04–3.43**
PLR, per 100 units	348	0.94	0.63–1.41
PLR ≥ 169.1	348	0.80	0.47–1.38
SII, per 100 units	348	1.02	0.96–1.07
SII ≥ 856	348	0.76	0.44–1.30
SIRI	361	1.11	0.93–1.33
SIRI ≥ 2.024	361	0.85	0.50–1.43
AISI, per 100 units	348	1.03	0.98–1.08
AISI ≥ 533.7	348	0.96	0.56–1.62

Note: Bold figures indicate statistical significance at the 0.05 level (1 is not included in the 95% CI).

AECOPD, acute exacerbation of chronic obstructive pulmonary disease; ICS, inhaled corticosteroids; LAMA, inhaled long-acting muscarinic antagonists; FEV_1, forced expiratory volume in one second; BMI, body mass index; CAT, COPD assessment test; aOR, adjusted odds ratio; CI, confidence interval; CRP, C-reactive protein; WBC, white blood cell count; PLR, platelet-to-lymphocyte ratio; SII, systemic immune-inflammation index; SIRI, systemic inflammation response index; AISI, aggregate index of systemic inflammation.
